# Molecular and Genetic Mechanisms That Mediate Transmission of *Yersinia pestis* by Fleas

**DOI:** 10.3390/biom11020210

**Published:** 2021-02-03

**Authors:** B. Joseph Hinnebusch, Clayton O. Jarrett, David M. Bland

**Affiliations:** Laboratory of Bacteriology, Rocky Mountain Laboratories, National Institute of Allergy and Infectious Diseases, National Institutes of Health, Hamilton, MT 59840, USA; claytonj@niaid.nih.gov (C.O.J.); david.bland@nih.gov (D.M.B.)

**Keywords:** *Yersinia pestis*, plague, fleas, arthropod-borne transmission

## Abstract

The ability to cause plague in mammals represents only half of the life history of *Yersinia pestis*. It is also able to colonize and produce a transmissible infection in the digestive tract of the flea, its insect host. Parallel to studies of the molecular mechanisms by which *Y. pestis* is able to overcome the immune response of its mammalian hosts, disseminate, and produce septicemia, studies of *Y. pestis*–flea interactions have led to the identification and characterization of important factors that lead to transmission by flea bite. *Y. pestis* adapts to the unique conditions in the flea gut by altering its metabolic physiology in ways that promote biofilm development, a common strategy by which bacteria cope with a nutrient-limited environment. Biofilm localization to the flea foregut disrupts normal fluid dynamics of blood feeding, resulting in regurgitative transmission. Many of the important genes, regulatory pathways, and molecules required for this process have been identified and are reviewed here.

## 1. Introduction

*Yersinia pestis* is a newly minted arthropod-borne pathogen, having adopted this lifestyle only within the last 3000 to 6000 years [1,2,3]. Due to its notorious virulence to humans and public health significance, most research on this pathogen justifiably has focused on the genetic and molecular mechanisms responsible for its pathogenicity in mammalian hosts. Its insect host, the flea, was proven to be the essential vector for *Y. pestis* transmission over 100 years ago; however, it has received sporadic and comparatively little attention over the years. Yet half of the life cycle and biology of *Y. pestis* can be said to occur in the insect host. From this standpoint, the term vector seems somewhat dismissive, as it may connote a simple conveyance from one mammalian host to the next. However, the ability to produce a transmissible infection in the flea is as critical to *Y. pestis* as its ability to produce a transmissible infection in the mammal. The two in fact are interrelated: reliance on the blood-feeding flea for transmission naturally selected for *Y. pestis* strains that produce a high-density bacteremia and, therefore, high virulence, in the mammal [4]. Thus, the study of the genetic and molecular mechanisms responsible for the ability of *Y. pestis* to infect and be transmitted by fleas is of interest from evolutionary, ecological, and general biology perspectives. It also has practical applications. As described below, *Y. pestis* develops a biochemically complex life stage in the flea that includes both bacterial and flea-derived components. It is in this unique form that the bacteria enter the mammal during a flea bite and first encounter the mammalian innate immune response.

## 2. The Insect Host Environment

**Figure 1:**
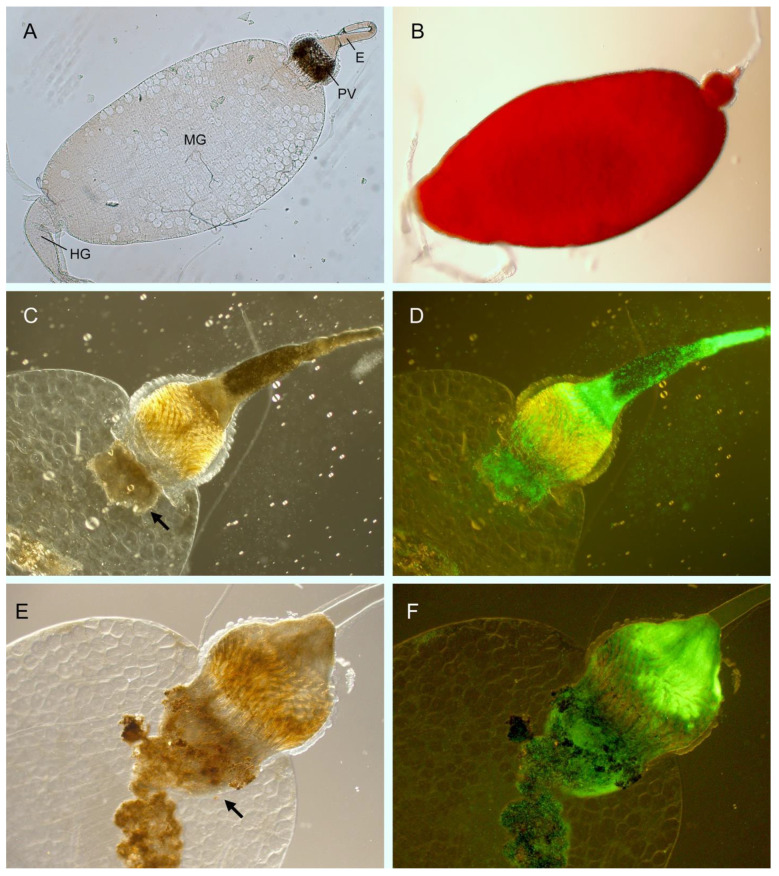
Anatomy of the flea digestive tract (E, esophagus; PV, proventriculus; MG, midgut; HG, hindgut) dissected and emptied of contents (**A**) or immediately after a blood meal (**B**). Middle row: digestive tract dissected three days after infection with *Y. pestis* expressing green fluorescent protein and photographed using light (**C**) or a combination of light and fluorescence (**D**) microscopy. Bottom row: digestive tract dissected from a flea with complete proventricular blockage (14 days after infection), photographed using light (**E**) or light and fluorescence (**F**) microscopy. Arrows point to the brown-colored, viscous biofilm extracellular polymeric substance (EPS) matrix in which the bacteria are enveloped.

Just as in the mammalian host, *Y. pestis* must sense specific environmental conditions and immune responses and adapt accordingly in order to successfully infect its insect host. The situation is less complicated in the insect, however, as *Y. pestis* remains confined to the lumen of the flea digestive tract. Infection does not involve any flea tissue or any other anatomical site. Fleas have a simple digestive tract, and storage, digestion, and absorption of the bloodmeal all occur in the unsegmented midgut (Figure 1A,B). The proventriculus, a heavily muscled valve, guards the entrance to the midgut, and its internal surface is arrayed with densely packed rows of inward-directed spines, which are coated with an acellular layer of cuticle. During blood feeding, the proventriculus rhythmically opens and closes in concert with contractions of the pharyngeal peristaltic pump muscles that impel blood into the midgut. After feeding, blood fills the digestive tract from the proventriculus to the hindgut and the midgut is significantly distended (Figure 1B). Digestion of the blood meal begins immediately, associated with peristaltic waves of the midgut and continued pulsations of the proventriculus, although the anterior end of the valve itself remains closed to retain blood in the midgut. This churning of the midgut contents and the threshing action of the proventricular spines presumably act to disaggregate red blood cells and mix them with digestive enzymes. Complete hemolysis and liquefaction of the blood meal occur rapidly, within only 3–6 h for female *Xenopsylla cheopis* fed on mouse blood [5,6]. Within a day, the blood meal has typically condensed into a dark brown, viscous residue.

Flea digestive tract physiology has been little studied, so few details are known about the chemical, physical, and other environmental conditions. The digestive tract is an open system, with periodic, recurrent input of fresh blood and excretion of digestion products. Some fleas, particularly those that feed frequently, such as the cat flea *Ctenocephalides felis*, excrete undigested and partially digested portions of their blood meal during or soon after feeding [7]. The midgut pH fluctuates between about 6.5 and 7 at different times after a blood meal, and osmolarity drops from around 500 to 300 mOsm, gradually increasing over the next 24 h to the original value as digestion proceeds [8]. The flea gut is aerobic, oxygenated by an extensive abdominal trachea system that is maximally active during blood digestion [9]. The brightness of oxygen-dependent green fluorescent protein molecules in the digestive tract is evidence of an aerobic environment.

Recently, genomics-based research is beginning to provide more specific insights into the insect host environment of *Y. pestis*. Transcriptomic profiling analyses revealed a large set of genes that were upregulated in the *X. cheopis* digestive tract tissue in response to a sterile or *Y. pestis*-infected blood meal [10]. Four hours after feeding, upregulated digestive enzyme genes most notably included several that encode serine proteases (trypsins or trypsin-like enzymes) and several enzymes of lipid digestion and metabolism pathways. This pattern fits with the biochemical composition of blood (95% protein and 4% lipid), but also points to blood lipids as being a major metabolic energy source for fleas.

Like all other insects, fleas initiate defense mechanisms against orally ingested microbes [11,12]. Early after infection with *Y. pestis*, the expression of several antibacterial effector genes is upregulated in *X. cheopis* digestive tract epithelia, including those encoding antimicrobial peptides and lysozyme. The response to *Y. pestis* appears to be due to upregulation of the immune deficiency (Imd) pathway, the arm of insect immunity directed against Gram-negative bacteria [10]. Another major arm of insect immunity is the dual oxidase-reactive oxygen species (Duox-ROS) system, which generates antibacterial oxygen radicals. *X. cheopis* fleas appear to mount this response, as several Duox-ROS pathway genes were upregulated following a blood meal containing *Y. pestis*, accompanied by increased hydrogen peroxide levels in the midgut [13]. Blood digestion itself produces oxidative stress by generating large amounts of heme, which can induce the formation of ROS [14,15]. The first genome sequence of a flea species was recently published, which should aid in further characterizing flea digestive tract physiology and the molecular mechanisms by which fleas respond to infection with *Y. pestis* [16].

## 3. Regurgitative Transmission of *Y. pestis* by Fleas

*Y. pestis* is a generalist, able to infect and be transmitted by many different flea species by the same general and rather crude mechanism. After entering the flea gut as single, planktonic, free-floating cells in a blood meal, a critical first step in colonization occurs within the first few hours, when many of the bacteria coalesce into large aggregates. These multicellular aggregates often extend from the proventricular valve into the midgut (Figure 1B,C). Fleas so colonized are able to transmit *Y. pestis* when they next feed again, a phenomenon referred to as early-phase transmission or mass transmission. In this case, the bacterial mass in the proventriculus is sufficient to partially and temporarily interfere with blood flow into the midgut, resulting in some backflow or regurgitation of blood containing dislodged bacteria into the bite site [17,18,19]. The obstruction is transient, however, as the bulk of the bacterial mass is readily flushed out of the proventriculus back into the midgut en masse by the incoming flow of blood. Following feeding, one part of the aggregate in the midgut can sometimes be seen to maintain the bulbous shape of the proventricular valve, ostensibly because it had become molded into a “cast” in its previous location [19]. This early-phase transmission was the first to be described and is relatively inefficient, as it requires several infected fleas feeding on an individual naïve host for successful transmission [20,21]. A second, related phase of transmission was subsequently described by Bacot and Martin, who observed that, with time, the bacterial aggregates could become more or less permanently entrenched in the proventriculus, with a high degree of resistance to being flushed back into the midgut during a blood meal [22]. In this case, blood feeding is partially or completely blocked by a bacterial mass that can completely fill the proventriculus (Figure 1E,F), and the ensuing hydrodynamic forces generated by continuous futile feeding act to reflux blood mixed with bacteria back into the bite site. In contrast to early-phase transmission, a single blocked flea has high transmission potential, particularly since it will make repeated feeding attempts over a period of a few days until it succumbs to starvation.

## 4. *Y. pestis* Transmission Factors

A major pursuit of microbial pathogenesis research has been the discovery of virulence factors, the biomolecules that are responsible for disease in vertebrates. The analogous biomolecules that are required to produce a transmissible infection in the invertebrate vector host have been termed transmission factors. Just as they do in the mammal, arthropod-borne pathogens must adapt to environmental conditions and antibacterial factors in their invertebrate hosts. They also often develop a particular phenotypic stage in a specific location in the arthropod that promotes their transmission. A shift from 37 °C to ambient, flea temperature by itself markedly affects *Y. pestis* gene expression in vitro [23,24]. Prominent virulence factors such as the F1 capsule and pH 6 antigens, iron acquisition operons, and RovA are downregulated [25]. Transcriptomic analyses of *Y. pestis* in mammalian host tissue and the flea digestive tract, as well as analyses of the flea transcriptomic response to oral infection with *Y. pestis*, have pointed out genes and gene regulatory systems that are induced in mammal or flea [10,13,25,26,27,28,29]. These have helped to lay the groundwork for much current research to identify and characterize the specific molecular interactions of *Y. pestis* with its insect host that lead to a transmissible infection.

### 4.1. Biomolecules Required to Survive and Grow in the Flea Digestive Tract: Resistance to the Flea Antibacterial Response

After entering the flea, *Y. pestis* experiences a rapid decrease in temperature and other changes in environment as the flea immediately begins to digest its blood meal. Digestion of host blood by fleas appears to generate a potent antibacterial product that is cytolytic to *Y. pestis* and other Gram-negative bacteria. A key biomolecule produced by *Y. pestis* is Ymt, a phospholipase D enzyme that acts to protect against the cytolytic agent [30] (Table 1). The *ymt* gene was acquired by *Y. pestis* after it diverged from *Y. pseudotuberculosis* by lateral gene transfer. Interestingly, the most closely related homologs of Ymt in GenBank have been found in other insect-associated Enterobacteriaceae, particularly *Arsenopholus* and *Photorhabdus* species. The *Y. pestis* Ymt protein is most similar to one from an *Arsenopholus* symbiont of an Asian insect pest of rice, consistent with phylogenetic evidence that *Y. pestis* first emerged in Asia.

**Figure 2:**
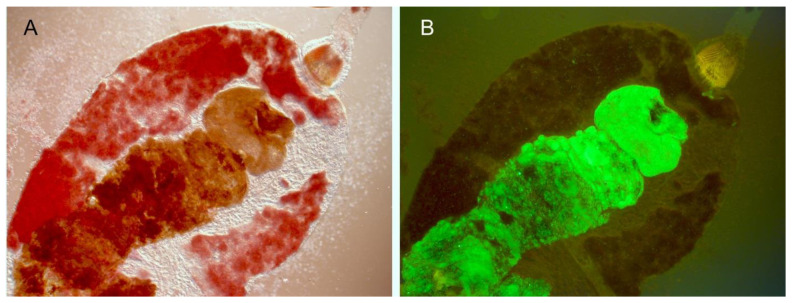
Digestive tract from a flea dissected one month after infection with *Y. pestis* expressing green fluorescent protein, photographed using bright field (**A**) and fluorescence (**B**) microscopy. This flea appeared healthy and took a normal blood meal even though its midgut was partially occupied by a large mass of *Y. pestis* biofilm.

The flea immune response to oral infection is analogous to the mammalian innate immune response, and the expression of several *Y. pestis* factors that protect against such a response is upregulated in the flea [10,27]. The PhoPQ two-component system (2CS) senses the decrease in pH experienced in the flea gut, which mediates lipid A modifications that protect against antimicrobial peptides [45]. Likely in response to the flea’s Duox-ROS system, the OxyR regulatory system protects against oxidative stress encountered in the flea, as a *Y. pestis oxyR* mutant is deficient for colonization [13]. On the other side, bacterial infection is potentially harmful to the health of the flea. It would be counterproductive if *Y. pestis* caused significant morbidity and mortality to its flea host before it was transmitted. Midgut infection with *Y. pestis* does not cause much morbidity, and fleas can remain infected and viable for months [59] (Figure 2). An important factor in this regard was the loss of the urease component gene *ureD* during the evolution of *Y. pestis* [60] (Table 2). The strong urease activity of the *Y. pseudotuberculosis* progenitor generates acute toxicity and high mortality of fleas soon after oral infection. Restoration of *ureD* in *Y. pestis* results in the same effect. Of course, once proventricular blockage develops, fleas starve to death within a few days. However, as noted previously, starvation actually enhances transmission because hungry and dehydrated blocked fleas make continued, persistent feeding attempts, each one with the possibility of regurgitative transmission.

### 4.2. Biomolecules Required to Survive and Grow in the Flea Digestive Tract: The Bacterial Autoaggregation Phenotype

The antibacterial effector pathways and molecules are important for resistance to the flea immune response and survival in the flea gut, but *Y. pestis* is also subject to clearance from the flea simply by elimination in flea feces. This is potentially a significant risk because *Y. pestis* does not adhere to flea digestive tract epithelia or proventricular spines. *Y. pestis* counteracts this by coalescing into large aggregates within a few hours after being ingested that localize and become enmeshed in the proventricular spines [17,19]. In addition to serving as a means to prevent elimination, aggregating bacteria are likely shielded from environmental stresses and antibacterial host responses. However, bacteria in the center of an aggregate may, for the same reason, be shielded from nutrients. Most examples of bacterial autoaggregation involve outer membrane proteins or adhesins [66], but to date no such involvement has been documented for *Y. pestis* in the flea. In fact, several adhesins of *Y. pseudotuberculosis* are absent or are pseudogenes in *Y. pestis*, including those encoding for InvA (invasin), YadA, YadE, Ifp/InvB, and Type IV pili [67,68]. Presumably, adhesion to and invasion of the flea digestive tract lining are detrimental because both would preclude regurgitative transmission. Bacterial fimbriae are also common adhesin molecules, and the *Y. pestis* genome contains nine gene clusters that encode intact chaperone/usher pathway fimbriae, including the Psa (pH 6 antigen) and Caf (F1 capsule) virulence factors. We have made deletions in six of these, but none of these individual mutants showed any deficiency in ability to colonize or block fleas [47], nor are any of the six essential for autoaggregation or biofilm formation in vitro [69]. The *Y. pestis* Ail outer surface protein can mediate autoaggregation in vitro, but an Ail mutant has a normal phenotype in the flea [70,71]. Exopolysaccharides can also be autoagglutinins, and the Hms polysaccharide is critical to the cohesiveness of *Y. pestis* aggregates in the flea gut; however, the large, dense bacterial aggregates form before the Hms exopolysaccharide is produced [17,19].

It is possible that redundant known or unknown *Y. pestis* autoagglutinins function to cause autoaggregation in the flea gut, but it is also possible that autoaggregation is passive, not mediated by bacterial factors but by physical conditions in the midgut. For example, simply transferring *Y. pestis* from pH 7 to pH 5.5 LB medium results in rapid autoaggregation [28]. Thus, the mechanism could involve surface electrostatic effects, such as hydrophobic interactions in an aqueous solution, or depletion interaction aggregation, a type of mechanically driven phase separation [66,72,73]. A non-bacterial substance present in the flea gut could also favor autoaggregation. For example, the bacterial aggregates appear to be surrounded by a brown, viscous, lipid- or waxy-appearing material, which may derive from blood lipid, protein and adsorbed hemin (Figure 1 and Figure 2).

### 4.3. Biomolecules Required to Survive and Grow in the Flea Digestive Tract: The Response to Nutrient Limitation and Other Stresses

Several lines of evidence indicate that the flea digestive tract is a physically stressful environment to Gram-negative bacteria. A transcriptional response is induced during infection of the flea gut that acts to protect *Y. pestis* to acid, osmotic, and other cell envelope stresses [25]. The PhoPQ gene regulatory system appears to be primarily induced by acid stress in the flea rather than by low Mg^2+^ ion concentration and modifies the bacterial outer membrane to confer resistance to insect antimicrobial peptides and presumably other cell envelope stressors [45]. If the PhoPQ system is nonfunctional, several other general and acid stress response genes are upregulated in the flea [28]. As the flea digests and absorbs blood meal elements, the osmolarity of the midgut contents increases [8], and several *Y. pestis* genes with osmoadaptive and osmoprotective functions are upregulated [25,27,29,74]. The stress-sensing alternative sigma factor, RpoE, is also upregulated in the flea [25].

*Y. pestis* is able to rapidly grow to large numbers in the flea gut after ingestion, often up to 10^6^ CFU per infected flea. Nevertheless, the midgut contents are apparently nutrient-restricted in some ways and require a substantial shift in bacterial metabolism. The gene expression pattern in the flea indicates that *Y. pestis* uses alternate carbon sources in the flea, particularly amino acids, rather than primary carbon sources, such as glucose [25,27], an adaptation that requires the cAMP receptor protein (CRP), the regulator of alternate carbon source metabolism and other cellular processes [48]. The gene for RovM, a transcriptional regulator of *Y. pseudotuberculosis* and *Y. pestis* that orchestrates the adaptive response to nutrient limitation in vitro, is highly upregulated in the flea [25,27]. A *Y. pestis rovM* mutant has a growth deficiency in the flea [31], another indication that the midgut is nutrient-limited.

Thus, although the oral ID_50_ dose required for *Y. pestis* to successfully produce a chronic infection in its flea host is high (a bacteremia level of ~10^7^ CFU/mL; [4,75]), and the flea gut a rather hostile environment for Gram-negative bacteria, *Y. pestis* is able to adapt, survive, and grow in that environment. Its numbers increase during the first week after an infectious blood meal and then plateau. However, in addition to intrinsic bacterial factors, it is important to note that characteristics of host blood can influence the infection and transmission efficiency of *Y. pestis* in fleas. For example, host bloods with a relatively insoluble hemoglobin molecule, such as rat blood, engender a more cohesive foregut infection that results in increased early-phase transmission efficiency [18]. Uncharacterized differences in flea species physiology also can influence vector competence—the infection rate of *Oropsylla montana*, a North American ground squirrel flea, is lower than that of *Xenopsylla cheopis* following an infectious mouse blood meal [76].

### 4.4. Biomolecules Required for Mature Biofilm Formation in the Flea Digestive Tract

A nearly universal adaptive response of bacteria to a stressful, growth-restricted environment is the development of a multicellular biofilm, which provides a protected and homeostatic niche that maximizes survival and persistence [77]. The *Y. pestis* life stage in its insect host is an example of this strategy. The initial autoaggregation and localization to the proventriculus can be seen as the first steps in this process. A mature biofilm, however, depends on a specific *Y. pestis* exopolysaccharide that is an important stabilizing component of the extracellular polymeric substance (EPS), or matrix, of the biofilm. The synthesis and export of this poly-β-1,6-*N*-acetyl-d-glucosamine exopolysaccharide is mediated by the *Y. pestis hmsHFRS* genes [36,64]. The Hms expolysaccharide is foundational for biofilm development in vitro and in the flea, and it is required for proventricular blockage in the flea [37,38].

Although the stresses, particularly nutrient limitation, that induce biofilm formation are common to many bacteria, the regulatory pathways and developmental programs are diverse and complex among different bacteria. For *Y. pestis*, two major regulatory systems that are induced in the flea (RovM and PhoPQ) have also been found to positively regulate biofilm development [32,78]. PhoPQ as well as another 2CS, OmpR-EnvZ, modify the bacterial outer surface to counteract environmental stresses, and these alterations are also important for biofilm formation. *Y. pestis* PhoP and OmpR-EnvZ mutants are able to infect fleas but are deficient in proventricular blocking ability [8,45], most likely due to lack of appropriate outer membrane modifications. For example, certain genes that affect LPS structure, such as *gmhA*, *yrbH*, and *waaA,* are required for a normal rate of flea blockage [51,56]. Ribose-P-isomerase, RpiA, is also required for optimal proventricular blockage [19]. The mechanism behind this is unknown, but also appears to be due an effect of RpiA on the *Y. pestis* outer membrane or biofilm EPS. From these results, it can be seen that *Y. pestis* outer surface characteristics, in addition to the Hms exopolysaccharide, are required for normal biofilm formation in the flea.

Another global regulator, CRP-cAMP, is critically involved in both metabolic adaptation to and biofilm development in the flea. CRP controls the carbon catabolite repression response by upregulating alternate carbon source metabolic pathways when glucose and other preferential carbohydrate sources are limited, which is the case in the flea midgut. The cAMP-CRP system appears to be a critical link between alternative carbon metabolism and biofilm development, as *Y. pestis* lacking CRP is deficient in biofilm formation in vitro [48,49]. Furthermore, although this mutant strain is able to survive in and produce a chronic infection in fleas, it never produces proventricular blockage [47].

Another layer of regulation of biofilm development occurs via small RNA molecules (sRNA). Two sRNAs, named HmsA and HmsB, have been implicated in activating biofilm development in vitro by upregulating the expression of the HmsCDE, HmsT, and HmsHFR genes [54,55]. A role for sRNA in biofilm development in the flea is also indicated by the fact that the Hfq RNA chaperone protein is required for proventricular blockage [52], although this may be due to a stabilizing effect that Hfq has on *hmsP* mRNA [53]. Another clue that sRNAs are involved in the regulation of biofilm development is that CsrA, the sRNA-binding protein component of the carbon storage system CsrABC, is required for normal Hms-exopolysaccharide production and biofilm development in vitro [48].

These complex and varied regulatory systems that upregulate biofilm formation in the flea function together to induce the Hms-exopolysaccharide, and other factors, essential for a cohesive EPS. In *Y. pestis* and many other Gram-negatives, the bacterial second messenger c-di-GMP is a critical molecule that induces a shift from planktonic to biofilm growth [79,80]. Intracellular levels of c-di-GMP in *Y. pestis* are determined by the relative activities of two diguanylate cyclase (DGC) enzymes, HmsT and Hms D, and one phosphodiesterase (PDE) enzyme, HmsP, that, respectively, synthesize and degrade c-di-GMP [39,40]. HmsD is the most active in the flea and is encoded in an operon that includes two flanking genes, *hmsC* and *hmsE*, which act to regulate *hmsD* expression [40,41,42,43]. These and other factors that act to promote Hms-exopolysaccharide and biofilm production are summarized in Table 1. Their complex interrelated regulatory patterns have been reviewed recently [32,78].

### 4.5. Biomolecules Left behind during the Evolution to Flea-Borne Transmission

The evolutionary change of *Y. pestis* to an arthropod-borne pathogen can be seen, in large part, as a fine-tuning of the preexisting biofilm development pathways of its *Y. pseudotuberculosis* ancestor to upregulate biofilm growth in, and transmissibility from, the flea gut. *Y. pseudotuberculosis* is able to form Hms-dependent biofilm in certain environmental conditions but does not do so in the flea [81]. In addition to gene gain (e.g., *ymt*), gene loss was important to this evolutionary process. For example, the Rcs multicomponent regulatory system inhibits biofilm development in *Yersinia* and other bacteria, and this system is highly expressed in the flea [25,62,82,83]. One of its components (*rcsA*), however, is a pseudogene in *Y. pestis*, thereby alleviating this negative regulation and increasing biofilm production in the flea [84]. In addition, two c-di-GMP degrading PDEs of *Y. pseudotuberculosis* were eliminated by gene loss in *Y. pestis* [39,40]. The importance of these genetic changes was demonstrated by introducing them into a *Y. pseudotuberculosis* strain, which then became capable of forming proventricular-blocking biofilm in fleas [61]. Gene losses that were important to adapting to the flea-borne route of transmission are summarized in Table 2.

## 5. Future Directions

Over 100 years after the seminal report of Bacot and Martin describing the proventricular blockage phenomenon and its importance for transmission, many molecular and genetic details have recently been discovered about how *Y. pestis* produces a transmissible infection in fleas. However, many missing pieces remain to be discovered. Although they both lead to c-di-GMP and Hms-exopolysaccharide production, the regulatory pathways differ in some ways between *Y. pestis* and *Y. pseudotuberculosis* that have yet to be explained. For example, RovM reportedly upregulates biofilm development in *Y. pestis* but represses it in *Y. pseudotuberculosis* by negatively regulating *hmsHFRS* [31,32,85]. In addition, biofilm formation in vitro and in the flea depends upon the expression of a Type 6 secretion system (T6SS) in *Y. pseudotuberculosis* but not in *Y. pestis* [29].

The effector mechanisms of the c-di-GMP signaling network have not yet been characterized. In other bacteria, these typically involve c-di-GMP-binding proteins and RNA molecules that interact with downstream targets to upregulate biofilm development [80]. Other unknowns include the molecular makeup of the *Y. pestis* biofilm EPS in the flea, much of which forms around *Y. pestis* in the flea before production of the Hms-exopolysaccharide [17,19]. The physiological state of *Y. pestis* during chronic infection of the flea in the biofilm state and the mechanism of bacterial release from the biofilm, an important element of transmission, have also not been investigated. Finally, although much can be learned from in vitro models of biofilm development, it will be essential to test the effect of implicated factors in the flea model, which cannot be duplicated by any in vitro conditions. Much work remains to be done to comparatively evaluate the vector competence of the many flea species implicated as vectors in the many ecological settings in which *Y. pestis* circulates. The biochemical characteristics of different host bloods and differences in flea-feeding behaviors, flea digestive tract physiology and proventricular anatomy all likely contribute to reported vector competence differences [17,18], but the mechanisms in large part have yet to be characterized.

## Figures and Tables

**Table 1:** *Y. pestis* biomolecules implicated in the ability to infect and be transmitted by fleas.

Name	Molecular Function	Role in the Flea	Reference
Ymt	phospholipase D	protection against toxic byproduct of blood digestion	[30]
RovM	transcriptional regulator	(i) metabolic adaptation to the flea gut; (ii) upregulates *hmsCDE*, *hmsT*; increases intracellular c-di-GMP and biofilm production	[31,32]
OxyR	transcriptional regulator	protection against reactive oxygen species generated in the flea	[13,33,34]
GalU, ArnB	lipid A modification enzymes	protection against antimicrobial peptides of flea immune response	[35]
HmsHFRS	glycolsyl transferase, polysaccharide deacetylase, transport porin	synthesis of extracellular β-1,6-GlcNAc polysaccharide biofilm matrix component	[36,37,38]
HmsCDE	diguanylate cyclase and its regulators	c-di-GMP synthesis	[39,40,41,42,43]
HmsT	diguanylate cyclase	c-di-GMP synthesis	[39,40]
HmsP	phosphodiesterase	c-di-GMP degradation	[44]
PhoP	transcriptional regulator	(i) protection against antimicrobial peptides of flea immune response; (ii) enhances stability of biofilm; mechanism unknown	[27,45,46]
Crp	cAMP receptor protein	(i) carbon catabolite regulation enabling metabolism of alternate carbon sources; (ii) required for biofilm production, activates *gmhA* and *waaAE-coaD*	[47,48,49]
YfbA	transcriptional regulator	enhances biofilm production; mechanism unknown	[50]
GmhA	phosphoheptose isomerase	heptose synthesis; LPS production	[51]
RpiA	ribose-5-phosphate isomerase	enhances stability of biofilm; mechanism unknown	[19]
Hfq	RNA chaperone	regulation of *hmsT* and *hmsP*	[52,53]
HmsA *	sRNA	upregulates *hmsCDE*, *hmsT*, *hmsHFRS;* increases intracellular c-di-GMP	[54]
HmsB *	sRNA	upregulates *hmsCDE*, *hmsT*, *hmsHFRS*; represses *hmsP;* increases intracellular c-di-GMP	[55]
CsrA *	carbon storage regulator protein	carbon catabolite regulation; enhances biofilm production; mechanism unknown	[48]
YrbH *	arabinose 5-phosphate isomerase	Kdo synthesis; LPS modification	[56]
WaaA *	Kdo transferase	Kdo synthesis; LPS modification	[56]
BfvR *	transcriptional regulator	upregulates *hmsHFRS*, *waaAE-coaD*, and *hmsCDE* expression	[57]
RpoZ *	RNA polymerase subunit	different variants affect rate of biofilm production; mechanism unknown	[58]

* Phenotypic effect demonstrated in vitro; not yet evaluated in the flea.

**Table 2:** Loss of function mutations in *Y. pestis* that favored biofilm production in the flea.

Name	Molecular Function	Original Role or Phenotype	Reference
PDE2 (Rtn)	phosphodiesterase	c-di-GMP degradation	[39,40,61]
PDE3	phosphodiesterase	c-di-GMP degradation	[39,40,61]
RcsA	transcriptional regulator	represses *hmsT, hmsCDE, hmsHFRS*; upregulates *hmsP*	[61,62,63]
UreD	urease enzyme subunit	urease generates toxic amounts of ammonia in the flea gut	[60]
NghA	glycosyl hydrolase	degradation of Hms exopolysaccharide	[64]
FlhD	regulator of flagellum synthesis	motility	[65]
